# Determination of Methylene Blue and Its Metabolite Residues in Aquatic Products by High-Performance Liquid Chromatography–Tandem Mass Spectrometry

**DOI:** 10.3390/molecules26164975

**Published:** 2021-08-17

**Authors:** Xuan Zhang, Yunhua Hui, Changling Fang, Yuan Wang, Feng Han, Xiaoyi Lou, Essy Kouadio Fodjo, Youqiong Cai, Cong Kong

**Affiliations:** 1 East China Sea Fisheries Research Institute, Chinese Academy of Fishery Sciences, Shanghai 200090, China; zhangxuan@ecsf.ac.cn (X.Z.); huiyunhuamaomao@126.com (Y.H.); fangling0334081@163.com (C.F.); wangyuan81@163.com (Y.W.); hanf@ecsf.ac.cn (F.H.); huoxingmayi@126.com (X.L.); 2 Key Laboratory of East China Sea Fisheries Research Institute, Chinese Academy of Fishery Sciences, Shanghai 200090, China; 3 Physical Chemistry Laboratory, UFR SSMT, Université Felix Houphouet Boigny, Abidjan 22 BP 582, Côte d’Ivoire; kouadio.essy@univ-fhb.edu.ci

**Keywords:** high-performance liquid chromatography–tandem mass spectrometry, methylene blue, disinfectant, aquatic products

## Abstract

A sensitive and reliable method was developed to determine methylene blue (MB) and its metabolite residues, including azure A (AZA), azure B (AZB), and azure C (AZC) in aquatic products by HPLC–MS/MS. The samples were extracted by acetonitrile and cleaned up by alumina-neutral (ALN) cartridges. The analytes were separated on a Sunfire C18 column (150 mm × 2.1 mm, 5 µm). The method was validated according to the European criteria of Commission Decision 2002/657/CE. Good linearity between 1–500 µg/L was obtained with correlation coefficients (*R*^2^) greater than 0.99. The limit of quantification (LOQ) was 1.0 µg/kg. The average recoveries at three levels of each compound (1, 5, and 10 µg/kg) were demonstrated to be in the range of 71.8–97.5%, with relative standard deviations (RSDs) from 1.05% to 8.63%. This method was suitable for the detection of methylene blue and its metabolite residues in aquatic products.

## 1. Introduction

Synthetic origin dyes are widely used in clothing, cosmetics, medicines, and even food because of their stability and low costs of production compared with natural source dyes. Dyes can be classified into anthraquinone, phenothiazine, and azo according to their structures. Methylene blue (MB) is a synthetic phenothiazine basic dye with pharmacological effects [1,2]. It was first used as a drug in the treatment of bacterial dysentery in 1891. MB can also be used as disinfectant to treat freshwater fish diseases of small melon worm disease, red mouth disease, oblique tube worm disease, etc. [3,4]. However, a high concentration of MB in aquatic products may cause toxic reactions to humans, such as nausea, vomiting, quadriplegia, and cyanosis [5,6]. It is reported that intravenous injection of 7 mg/kg MB can cause severe gastrointestinal diseases in adults [7]. Since malachite green, crystal violet, and other triphenylmethane dyes were banned in aquaculture, MB has become a better substitute. The use of MB in aquaculture is prohibited in many countries, including the USA, the European Union, and Japan [8,9,10]. However, it is still used in aquaculture for disease treatment, water disinfection, and transportation [11].

**Figure 1:**
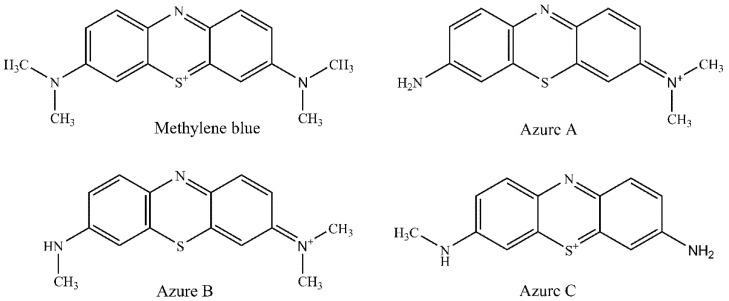
Chemical structures of methylene blue, azure A, azure B, and azure C.

MB could be metabolized by a demethylation process into azure A (AZA), azure B (AZB), and azure C (AZC) via, which are easy to dissolve in water and alcohol according to their chemical structures (Figure 1). The determination of MB and its metabolite residues in aquatic products is vital for food safety. Recent studies mostly focused on the removal of MB but less on the simultaneous determination of MB and its metabolite residues in food [12,13]. Lately, various detection methods such as spectrophotometry [6,14], indirect competitive enzyme-linked immunosorbent assay (IC-ELISA) [15], resonance light scattering (RLS) [16], liquid chromatography (LC) [8], and liquid chromatography–tandem mass spectrometry (LC–MS/MS) [17,18] for MB and its metabolite residues in aquatic products have been developed. As the LC method is sensitive, rapid, and accurate, it has become the most used method for the detection of MB in food. A green approach for the determination of MB in channel catfish tissue by HPLC was proposed, and the LOD was 10 µg/kg [8]. Yu et al. established a new LC–MS/MS method for the determination of MB and its metabolite residues in fish tissue with an LOQ of 2.0 µg/kg [19]. In addition, Xu et al. [14] developed a surface-enhanced Raman spectroscopy (SERS) method to detect MB in four fish tissues with the limit of detection (LOD) of 1–5 µg/kg. Methods for the analysis of phenothiazine dyes in animal tissues have been limited to MB and AZB. In this study, a sensitive and reliable method for the simultaneous determination of MB and its metabolites (AZA, AZB and AZC) using LC–MS/MS was developed. This method uses acetonitrile for extraction and alumina-neutral (ALN) cartridges for clean-up, which provides the benefits of versatility, high extraction efficiency, and a stable extraction process. Its analytical performance was validated in terms of linearity, limit of detection, limit of quantification, and intraday and inter-day precision. Its application in MB and its metabolites (AZA, AZB, and AZC) analysis in aquatic products was demonstrated.

## 2. Results and Discussion

### 2.1. HPLC–MS/MS Conditions

To optimize the mass characterizations, direct infusion of individual standard solution (100 µg/L) of each compound was performed. Mass scans were performed in positive ion mode with the flow rate of 10 µL/min. Two characteristic fragments of each compound were selected for qualitative and quantitative purposes; ionization parameters and collision energy were optimized at the end.

**Figure 2:**
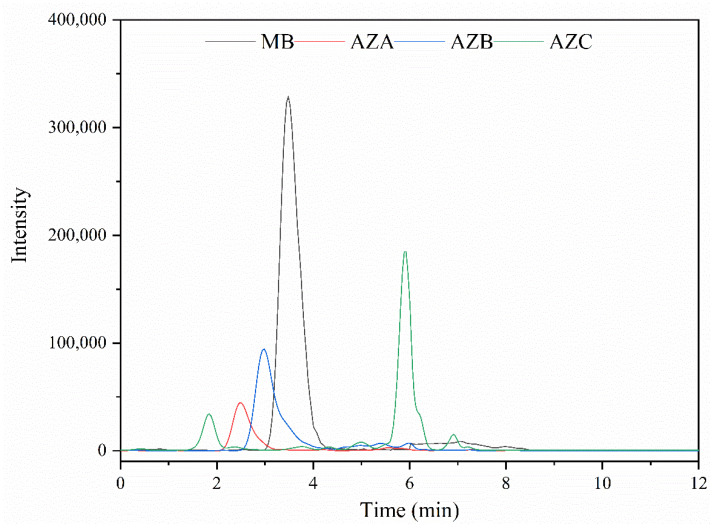
Chromatographic peaks of MB, AZA, AZB, and AZC.

Due to the properties of the compounds under study, C_18_ chromatographic columns were chosen for the separation [20]. In this study, four C_18_ columns, including Sunfire C_18_ column, Symmetry C_18_ column, ODS C_18_ column, and Capcell Pak C_18_ column, were compared, and the results show that the Waters Sunfire C_18_ column presented good separation. The mobile phases were composed of acetonitrile, methanol, and ammonium acetate buffer (0.005 M) acidified with 0.2% formic acid. The methanol could strengthen the retention of AZC on C_18_ column. Moreover, the addition of ammonium acetate and formic acid could result in better chromatographic peak shapes. Figure 2 shows the total ion chromatogram of the four analytes after LC optimization. Figure S1 shows the chromatogram of bream blank sample spiked with 10 μg/kg (see Supplementary Materials). The retention time, fragment information, and collision energies are listed in Table 1.

### 2.2. Method Development

Due to the low content of the studied analytes in aquatic products, the extraction process is essential. Liquid–liquid extraction (LLE) [18,20], liquid-phase microextraction (LPME) [3], and solid-phase microextraction (SPME) [4] are usually used for extraction. Extraction agents including acetonitrile, acetonitrile/buffer solution, and other polarity organic reagents [3] are commonly used. The proper extraction method can dissolve target compounds and precipitate protein. Thus, the extraction process of the developed method was operated through a liquid–liquid extraction using acetonitrile as an extraction solvent. Acetonitrile/acetone (1:1, *v*/*v*) and acetonitrile/ethyl alcohol (1:1, *v*/*v*) were also tested, but the acetonitrile was selected as the final extraction solution due to its high extract efficiency for all target analytes and good performance in protein precipitation.

**Figure 3:**
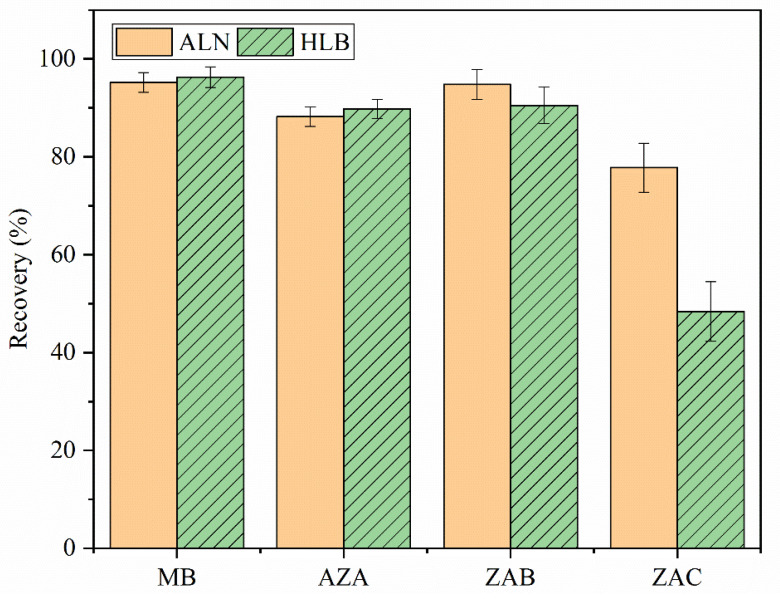
Recoveries of MB, AZA, AZB, and AZC after clean-up with ALN and HLB cartridges on the extract from crucian carp samples (*n* = 6).

The fish samples were a complex matrix (containing water, fat, protein, and colored compounds). Accordingly, a clean-up process needed to be performed before HPLC–MS/MS analysis. As reported, there are many types of clean-up approaches, including the liquid–liquid distribution method [19], dispersed solid-phase method [14], and solid-phase extraction cartridge method [20,21]. The synthetic phenothiazine basic dyes we studied here are cationic compounds. Hence ALN cartridges and hydrophilic–lipophilic balanced (HLB) cartridges as the most common cation exchange cartridges in the laboratory were tested for clean-up by spiking in crucian carp samples with 5 µg/kg (Figure 3). Acceptable recoveries were achieved using HLB cartridges, except for AZC (48.4%), which were lower than using ALN cartridges (77.8%). The recoveries of all analytes ranged from 77.8% to 95.2% using ALN cartridges, which indicates that ALN cartridges could result in better removal of impurities (fat, water, colored compounds, etc.) and less target substance loss. Therefore, ALN cartridges were chosen as the final clean-up agent.

### 2.3. Method Validation

Method specificity was validated by testing 28 samples of different aquatic products (bream, trout, grass carp, fresh shrimps, Chinese mitten crab, crucian carp, perch, catfish, and mussel). No potential interfering peaks on the retention times of the analytes were observed. Seven concentration levels were prepared for linear range (LR), limit of detection (LOD), and limit of quantification (LOQ) (Table 2). Good linearity of each compound with a linearity (*R*^2^) of greater than 0.99 was obtained. The LOD and LOQ were 0.75 µg/kg and 1.0 µg/kg, respectively. These results suggest that this method is more sensitive than the reported method using HPLC–MS/MS for the determination of MB and its metabolite residues in aquatic products [18,19,20].

The matrix effect (ME) was evaluated in different fish samples (bream, fresh shrimps, Chinese mitten crab, catfish, and mussel). Table 2 lists the average ME of all the analytes. The results show ionization suppression of 2.9–15.9% for target analytes, which indicates that no significant matrix effect was observed for all kinds of matrices.

The stability and reproducibility of the proposed method were established. For stability, different fish samples of bream, trout, grass carp, fresh shrimps, Chinese mitten crab, crucian carp, perch, catfish, and mussel were tested, and the RSDs of inter-day precision ranged from 5.61% to 9.59%. For the reproducibility, three replicate grass carp samples spiked with mixture standards were extracted and cleaned up for LC–MS/MS analysis. The relative standard deviations (RSDs) of intraday variation ranged from 4.34% to 7.08%. These results show acceptable stability and reproducibility of the developed method.

For the method accuracy and RSDs of precision, three concentration levels of standards were spiked in various fish samples (bream, trout, grass carp, fresh shrimps, Chinese mitten crab, crucian carp, perch, catfish, and mussel). The recoveries ranged from 71.8% to 97.5%, with the RSDs of precision ranging between 1.05% and 8.63%, indicating good accuracy and precision of the described method (Table 3).

Table 4 lists organic extraction solvent, purification agent, sample matrix, number of the analytes, LR, LOQ, RSD, and recovery of reported LC–MS/MS methods in the determination of MB and its metabolite residues in aquatic products. This LC–MS/MS method we developed offered high specificity and separation efficiency with good stability and reproducibility for the determination of MB and its metabolite residues in various kinds of aquatic products. Compared with the method performed by Amelin et al. (the LOQ of AZA, AZB, and AZC was 4 µg/kg) [18], the present method had a lower LOQ of AZA, AZB, and AZC. This method covers a wider range of aquatic products. The pretreatment of the method is simple and easy to operate, as the extraction samples are only cleaned once [19] and no oxidation reaction is required [21]. The established method provides similar accuracy, with the advantages of being sensitive and precise in the simultaneous determination of the four analytes.

### 2.4. Application of the Method

Bathed fresh crucian carp samples in 10 mg/L MB for 0.5 h, then washed and kept the fish in clean water for an hour. The concentration levels of MB, AZA, AZB, and AZC in muscle were determined using the proposed method and reported method according to Yu et al. [19], respectively. The residue for MB at 41.3 µg/kg, AZA at 18.0 µg/kg, and AZB at 73.6 µg/kg were detected using proposed method and no AZC was detected. Similar results were obtained for MB at 42.7 µg/kg, AZA at 19.1 µg/kg, and AZB at 74.9 µg/kg using the reported method. In addition, 42 samples (including 10 crucian carp samples, 10 catfish samples, 10 fresh shrimps, 3 crab samples, and 9 mussel samples) were tested, which were obtained from Shanghai local markets, and no MB and its metabolite residues were detected. Thus, the established method can be used to assess the exposure of aquatic products to MB and its metabolite residues.

## 3. Materials and Methods

### 3.1. Materials

MB (C_16_H_18_ClN_3_S, 98%) and AZB (C_15_H_16_ClN_3_S, 98%) were purchased from Sigma-Aldrich (St. Louis, MO, USA). AZA (C_14_H_14_ClN_3_S, 80%) and AZC (C_13_H_12_ClN_3_S, 50%) were purchased from West Asia Chemical Industry Co. Ltd. (Shandong, China). Methanol and acetonitrile (HPLC grade) were obtained from J. T. Baker Chemical Co. (Phillipsburg, NJ, USA). Polytetrafluoroethylene (PTFE, 0.22 µm) filters were supplied by Branch Billion Lung Experimental Equipment Co., Ltd. (Tianjin, China). Ammonium acetate (HPLC grade) was obtained from Honeywell (Morris Plains, NJ, USA). Formic acid (HPLC grade) was supplied by Aladdin Biochemical Technology Co., Ltd. (Shanghai, China). The ultrapure water was produced using Milli-Q Pure Water System (Millipore, Billerica, MA, USA). Alumina-neutral (ALN) solid-phase extraction (SPE) cartridges (3 mL/500 mg) were purchased from Sigma-Aldrich (St. Louis, MO, USA). Hydrophilic–lipophilic balanced (HLB) cartridges (6 mL/200 mg) were supplied by Waters (Taunton, MA, USA). The columns were Sunfire C_18_ column (150 mm × 2.1 mm, 5 µm, Waters Technologies Ltd., Wexford, Ireland), Symmetry C_18_ column (100 mm × 2.1 mm,3.5 µm, Waters Technologies Ltd., Wexford, Ireland), Hypersil ODS C_18_ column (150 mm × 2.1 mm, 5 μm, Thermo Fisher Scientific Ltd., Bartlesville, OK, USA), and Capcell Pak C_18_ column (100 mm × 2 mm, 5 µm, Shiseido Ltd., Tokyo, Japan).

### 3.2. Instrumentation and Chromatographic Conditions

The triple-quadrupole mass spectrometer TSQ Quantum Access Max (Thermo Fisher Scientific, Bremen, Germany) coupled to the Surveyor high-performance liquid chromatography system (Thermo Fisher Scientific, San Jose, CA, USA) was used. The separation was obtained on a Sunfire C_18_ column (150 mm × 2.1 mm, 5 µm, Waters Technologies Ltd., Wexford, Ireland). The flow rate was set at 0.3 mL/min, with a sample injection volume of 25 µL and the column temperature at 30 °C. Mobile phase A was ammonium acetate buffer (0.005 M) acidified with 0.2% formic acid, mobile phase B was methanol, and mobile phase C was acetonitrile. The LC system was equilibrated for 30 min before the analysis. The gradient program is listed in Table 5.

Selected reaction monitoring (SRM) mode was acquired and processed in electrospray interface (ESI) positive mode. The ionization parameters were as follows: needle spray voltage, 4 kV; sheath gas pressure (N_2_), 30 arbitrary units; auxiliary gas pressure (N_2_), 5 arbitrary units; ion transfer capillary temperature, 300 °C; collision gas pressure (Ar), 1.5 mTorr. The retention time, fragment information, and collision energies are displayed in Table 1.

### 3.3. Standard Stock Solutions Preparation

Individual stock solution of each analyte was prepared at 100 mg/L by accurately dissolving an appropriate amount of the standard compound in methanol. Mixed intermediate solution at 1 mg/L was prepared by diluting standard stock solutions in methanol. All these standard solutions were stocked in ambered flasks at −18 °C and were found stable for at least 4 weeks. All mixed working standard solutions ranging from 1 µg/L to 500 µg/L (at seven gradient concentration levels) were prepared fresh daily by diluting the intermediate solution in methanol.

### 3.4. Method Validation

The LC–MS/MS method was validated according to the Commission Decision 2002/657/CE [23]. The LOD and LOQ were established from the calibration curve in accordance with the ISO standard 11843 [24]. The qualitative analysis was verified by relative ion intensities and retention time. The quantitative analysis was performed using a linear regression model relating concentrations and the quantitative ion intensities from analytes. The parameters of the method were validated in terms of specificity, *R*^2^, LR, LOD, LOQ, precision (repeatability and reproducibility), recovery, matrix effects, and stability.

For the specificity, at least 28 samples of different aquatic products (bream, trout, grass carp, fresh shrimps, Chinese mitten crab, crucian carp, perch, catfish, and mussel) were tested in order to check the presence of potential interfering peaks at the retention times of the analytes.

The ionization of target analytes can be affected by matrix components, which will reduce or enhance the intensities of the analytes. Thus, the ME of the proposed method must be studied. For the matrix effect, two sets of analytical curves were analyzed. One was the solvent standard curve, and the other one was the matrix-matched standard curve [25]. The effect of endogenous compounds in sample matrix on signal intensity of LC–MS/MS was checked. ME was calculated as follows:

ME = (1 − slope of matrix-matched standard curve/slope of solvent standard curve) × 100(1)

Values of ME more than 0 indicate ionization suppression, while values lower than 0 indicate ionization enhancement. It is considered to be minor when the values of ME are less than ±20% [26]. ME for different aquatic products were assessed, including bream, fresh shrimps, Chinese mitten crab, catfish, and mussel. The matrix-matched curves were obtained by spiking standard solutions after the evaporation process.

Linear ranges of all the compounds ranged from 1 µg/L to 500 µg/L (at seven concentration levels). The method linearity was established with three repetitions in two days. Linearity was established using a linear regression model relating concentrations and the signal intensities from each analyte. For method accuracy and RSDs of precision, six replicates of spiked samples (bream, trout, grass carp, fresh shrimps, Chinese mitten crab, crucian carp, perch, catfish, and mussel) at three levels of each compound (1, 5, and 10 µg/kg) were analyzed.

The stability of processed samples was evaluated in triplicate on three consecutive days of cold storage at −18 °C. For reproducibility (RSDs of intraday precision), three replicate grass carp samples spiked with mixture standard were extracted, cleaned up, and injected in LC–MS/MS.

### 3.5. Sample Collection and Sample Preparation

All the fish samples were obtained from Shanghai local markets. Edible parts of the fish were homogenized by a blender and stored at −18 °C.

Amounts of 5.00 (±0.05) g of homogeneous samples were weighed and transferred into a 50 mL polypropylene centrifuge tube. After adding 11 mL acetonitrile, the samples were vortexed for 30 s using Vortex Mixer (IKA, Staufen, Germany). The homogenate was placed for 10 min at room temperature under an ultrasonic bath (3510-DTH, Branson, Connecticut, USA). After centrifugation at 2150× *g* for 10 min (CF16RX, Hitachi, Tokyo, Japan), the supernatant was collected into a 25 mL calibrated flask. The residues were re-extracted with 11 mL acetonitrile as before. All the supernatants were combined in the calibrated flask and diluted to a final volume of 25 mL with acetonitrile. Then the supernatants were shaken for 1 min for further purification on ALN cartridges.

The cartridge was used in matrix adsorption mode to remove interferences, and the analytes of interest flowed through the cartridge during the loading step. The ALN cartridge was conditioned with 5 mL of acetonitrile. Then, 5 mL of the supernatant was passed through the cartridge, which was rinsed with 5 mL of acetonitrile and blown to dry from the top with a rubber suction bulb. All the elution was combined into a clean tube and dried under nitrogen gas at 38 °C. The residue was dissolved in 1 mL of a solution of ammonium acetate buffer (0.005 M) acidified with 0.2% formic acid/methanol/acetonitrile (63:22:15, *v*/*v*/*v*). The final solution was vortex-mixed, filtered through a PTFE (0.22 µm) filter, and transferred into a vial for LC–MS/MS analysis.

### 3.6. Statistical Analysis

All samples were carried out with three replicates and data are expressed as means ± standard deviations. The SPSS 16.0 software package (SPSS Inc., Chicago, IL, USA) was used, with *p* < 0.05 considered to indicate statistical significance. Analysis of variance (ANOVA) followed by Duncan’s test was used to test the significance of differences.

## 4. Conclusions

In summary, a sensitive and reliable method for the determination of MB and its metabolite residues in aquatic products by high-performance liquid chromatography with electrospray ionization tandem mass spectrometry was established. Liquid–liquid extraction and ALN cartridges were used for sample preparation. The developed method was validated according to the Commission Decision 2002/657/CE, with good accuracy, stability, reproducibility, and sensitivity; the LODs and LOQs were 0.75 µg/kg and 1.0 µg/kg, respectively. Moreover, we successfully applied the method in the analysis of various aquatic products. The recoveries were in the range of 71.8–97.5%, with the RSDs in the range of 1.05–8.63%. This method could be a suitable alternative for monitoring MB and its metabolite residues in aquatic products.

## Figures and Tables

**Table 1:** MS parameters of the four compounds.

Compounds	Retention Time (min)	Q1 Mass (*m*/*z*)	Q3 Mass (*m*/*z*)	Collision Energy (eV)	Ion Ratio (%)
MB	3.48	284.0	268.1 ^1^/252.0	34/49	28
AZA	2.49	256.1	241.0 ^1^/199.0	32/33	70
AZB	2.98	270.1	254.1 ^1^/212.0	34/51	30
AZC	1.84	242.1	200.1 ^1^/227.0	33/26	34

^1^ Quantitative ion.

**Table 2:** Quantitative performance of the developed method.

Compounds	LR (µg/kg)	*R* ^2^	LOD (µg/kg)	LOQ (µg/kg)	Intraday RSD (*n* = 3) (%)	Inter-Day RSD (*n* = 3, day) (%)	ME (%)
MB	1–500	0.9982	0.75	1.0	4.95	6.47	2.9
AZA	1–500	0.9966	0.75	1.0	6.75	8.50	12.8
AZB	1–500	0.9978	0.75	1.0	4.34	5.61	7.0
AZC	1–500	0.9959	0.75	1.0	7.08	9.59	15.9

**Table 3:** Recoveries and precision of four compounds spiked in different aquatic products (*n* = 3).

Analytes	Add Level(µg/kg)	Recovery/% (RSD/%)								
		Bream	Trout	Grass Carp	Fresh Shrimps	Chinese Mitten Crab	Crucian Carp	Perch	Catfish	Mussel
MB	1	93.2 (2.63)	96.5 (4.27)	95.2 (4.51)	96.7 (7.04)	89.9 (6.58)	92.7 (3.04)	91.5 (2.21)	90.5 (4.18)	89.7 (6.24)
	5	90.5 (5.23)	95.0 (4.01)	93.4 (3.28)	95.1 (3.24)	91.1 (5.63)	93.3 (5.32)	93.5 (3.08)	96.0 (5.36)	90.5 (6.06)
	10	96.4 (6.37)	95.3 (7.68)	92.5 (4.05)	97.5 (2.84)	90.7 (6.70)	90.5 (5.01)	93.9 (4.29)	93.2 (1.05)	91.5 (5.71)
AZA	1	86.2 (7.15)	82.9 (7.52)	88.9 (3.57)	87.2 (6.35)	83.2 (8.04)	86.1 (3.82)	88.6 (3.66)	83.5 (6.01)	81.6 (1.28)
	5	88.3 (8.63)	84.9 (7.67)	90.7 (3.69)	84.9 (5.08)	86.4 (8.36)	83.1 (3.66)	87.9 (2.08)	85.5 (5.47)	82.0 (1.38)
	10	91.0 (5.72)	89.5 (5.34)	91.0 (2.61)	86.5 (7.04)	88.7 (6.04)	88.6 (2.79)	89.2 (1.99)	83.9 (3.28)	85.0 (1.98)
AZB	1	95.3 (4.25)	96.0 (5.05)	97.1 (1.07)	95.4 (5.60)	92.5 (7.74)	94.3 (1.35)	92.8 (5.27)	91.8 (2.04)	93.4 (3.06)
	5	96.0 (3.35)	94.2 (4.75)	95.0 (2.67)	96.2 (5.07)	93.4 (6.66)	90.5 (2.07)	90.8 (3.31)	93.0 (1.29)	96.0 (1.08)
	10	94.2 (3.07)	93.8 (5.01)	93.8 (1.96)	93.7 (4.14)	95.2 (4.24)	92.0 (1.72)	95.4 (3.07)	91.2 (2.22)	94.4 (1.66)
AZC	1	73.2 (3.58)	75.1 (5.05)	74.5 (3.77)	72.1 (5.74)	71.8 (8.05)	75.4 (4.76)	74.8 (6.12)	75.5 (7.22)	72.1 (6.01)
	5	72.8 (4.57)	77.0 (5.34)	74.3 (3.04)	75.4 (4.87)	72.2 (6.80)	73.5 (5.33)	73.0 (5.76)	72.0 (2.05)	74.2 (5.20)
	10	75.7 (3.39)	78.7 (1.87)	72.7 (3.30)	72.7 (5.02)	78.1 (6.17)	78.6 (4.21)	72.8 (3.35)	74.6 (5.00)	76.6 (6.45)

**Table 4:** Comparison of the present method with other reported methods for determination of methylene blue and its metabolite residues in aquatic products.

Extraction Organic Solvent	Purification Agent	Sample	N ^1^	LR (µg/L)	LOQ (µg/kg)	RSD (%)	Recovery (%)	Ref.
Acetonitrile	ALN cartridge	Aquatic products	4	1–500	1.0	1.05–8.63	71.8–97.5	Present method
Acetonitrile containing 0.1% of formic acid	-	Aquatic products	4	0.04–2.0	0.4–4	5–15	87–130	[18]
Ammonium acetate buffer solution and acetonitrile	Liquid–liquid distribution and PRS ^2^ cartridge	Aquatic products	4	1–100	2	2.3–13.0	70.3–92.1	[19]
Acetonitrile	-	Plasma	2	1–1000	30	3.6–11.9	97.9–108.0	[20]
Acetonitrile	CBA and SCX-SPE ^3^ cartridges	Eel	2	2–16	0.25	8.3–14.5	84.1–102.1	[21]
Acetonitrile/sodium acetate buffer solution	ALN cartridge	Eel, toasted eel, and shrimp	1	2.0–100.0	0.5	4.4–16.3	73.0–108.3	[22]

^1^ Number of analytes. ^2^ Propanesulfonic acid bonded. ^3^ Weak cation exchange cartridges and strong cation exchange cartridges-solid phase extraction.

**Table 5:** Gradient elution program of the target analytes.

Time (min)	Mobile Phase A (%)	Mobile Phase B (%)	Mobile Phase C (%)
0.0	63	22	15
2.4	63	22	15
2.5	28	42	30
7.0	28	42	30
7.1	0	60	40
15.0	0	60	40
15.1	63	22	15
18.0	63	22	15

## Data Availability

The data presented in this study are available on request from the corresponding author.

## Supplementary Materials

The following are available online, Figure S1: Chromatogram of bream blank sample spiked with 10 μg/kg.

## Sample Availability

Samples of the compounds are not available from the authors.
