# Supplementary material for: Determination of Methylene Blue and Its Metabolite Residues in Aquatic Products by High-Performance Liquid Chromatography–Tandem Mass Spectrometry
Source: Molecules. 2021 Aug 17;26(16):4975. doi: 10.3390/molecules26164975 (PMC8401997; doi:10.3390/molecules26164975)
Supplement: Supplementary file 1 [file molecules-26-04975-s001.zip › molecules-1332950-supplementary.pdf]

Supplementary Materials

Determination of Methylene Blue and its Metabolite Residues in Aquatic Products by High-Performance Liquid Chromatography–Tandem Mass Spectrometry

Xuan Zhang [1,2,†], Yunhua Hui [1,†], Changling Fang [1,2], Yuan Wang [1], Feng Han [1,2], Xiaoyi Lou [1,2], Essy Kouadio Fodjo [3], Youqiong Cai [1,*] and Cong Kong [1,2,*]

[1] East China Sea Fisheries Research Institute, Chinese Academy of Fishery Sciences, Shanghai 200090, China; zhangxuan@ecsf.ac.cn (X.Z.); huiyunhuamaomao@126.com (Y.H.); fangling0334081@163.com (C.F.); wangyuan81@163.com (Y.W.); hanf@ecsf.ac.cn (F.H.); huoxingmayi@126.com (X.L.)
[2] Key Laboratory of East China Sea Fisheries Research Institute, Chinese Academy of Fishery Sciences, Shanghai 200090, China
[3] Physical Chemistry Laboratory, UFR SSMT, Université Felix Houphouet Boigny, 22 BP 582 Abidjan 22, Ivory Coast; kouadio.essy@univ-fhb.edu.ci
* Correspondence: caiyouqiong@163.com (Y.C.); kongc@ecsf.ac.cn (C.K.); Tel.: +86-021-6568-4297 (Y.C.); +86-021-6568-0121 (C.K.)
† These authors contributed equally to this work.

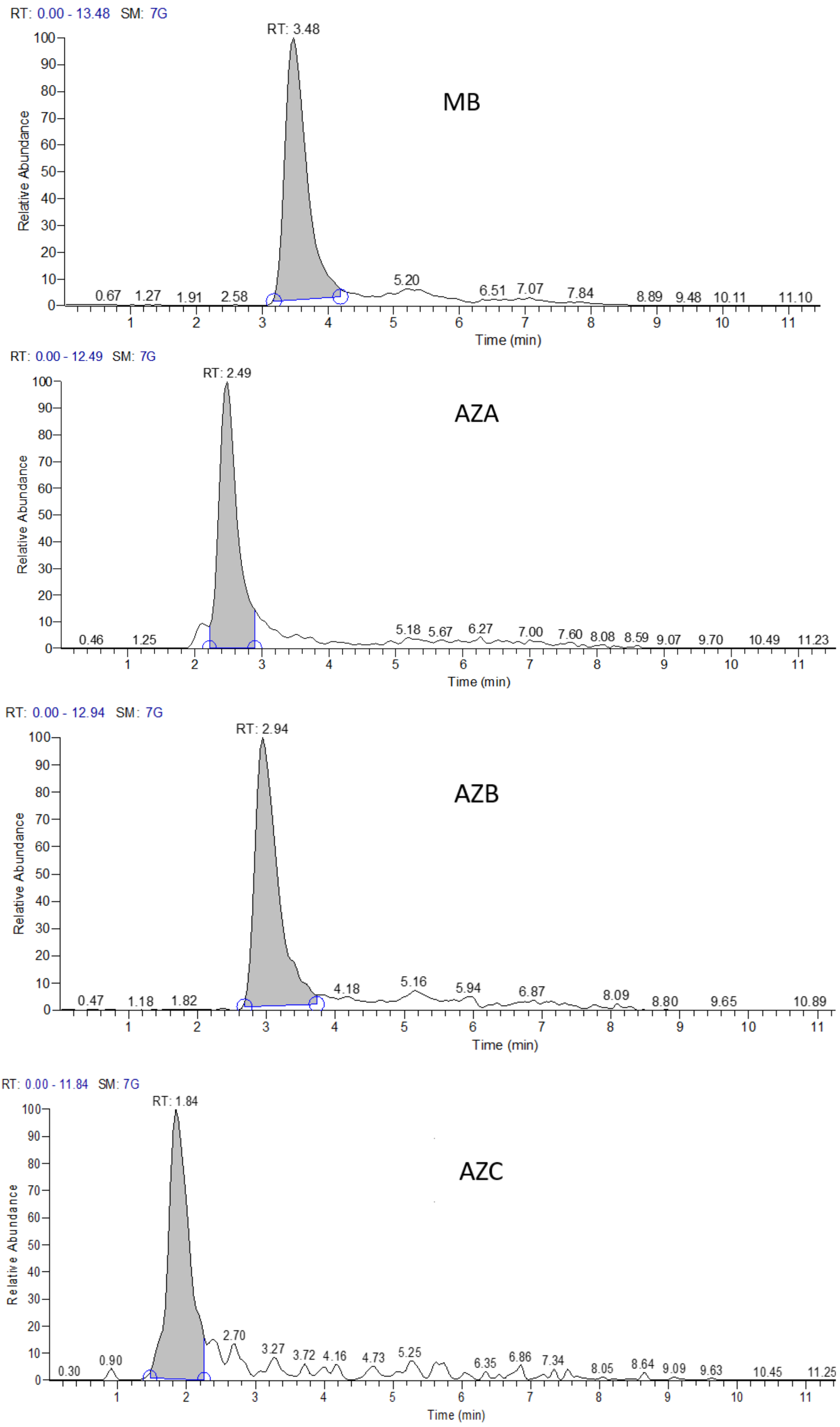

Figure S1. Chromatogram of bream blank sample spiked with 10 µg/kg.
